# Supplementary material for: Induction of PARP7 Creates a Vulnerability for Growth Inhibition by RBN2397 in Prostate Cancer Cells
Source: Cancer Res Commun. 2023 Apr 17;3(4):592–606. doi: 10.1158/2767-9764.CRC-23-0086 (PMC10108886; doi:10.1158/2767-9764.CRC-23-0086)
Supplement: Supplementary Figure 1 — Cell cycle distributions of cell lines treated with RBN2397 plus androgen or plus AHR agonist [file crc-23-0086-s02.pdf]

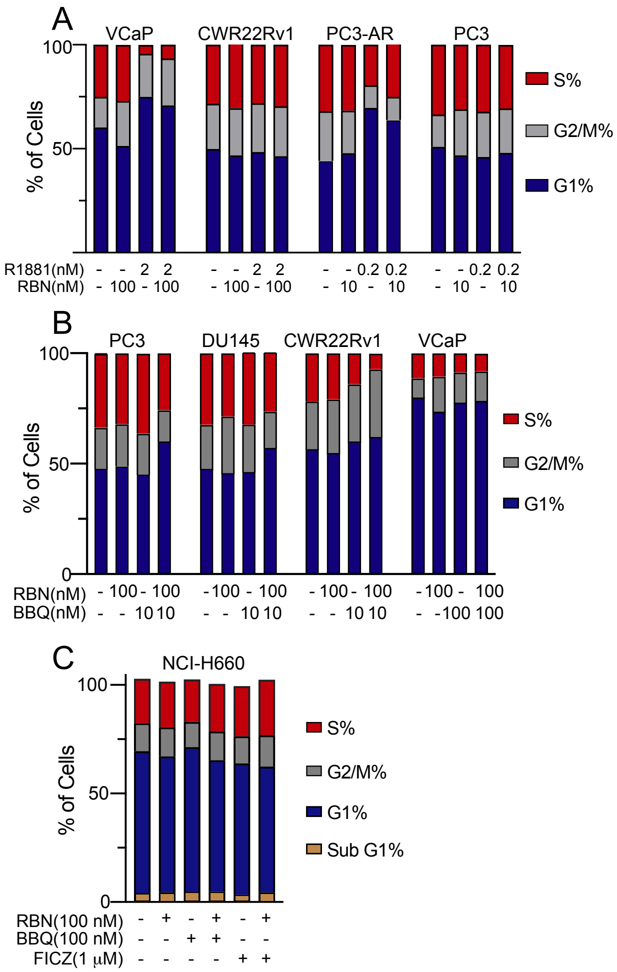

Supplementary Figure 1. Cell cycle distributions of cell lines treated with RBN2397 plus androgen (A) or plus AHR agonist (B, C).

(A) Data from VCaP, CWR22Rv1, PC3-AR and PC3 cells treated with the combinations of R1881 and RBN2397 as indicated.

(B) Data from PC3, DU145, CWR22Rv1 and VCaP cells treated with the combinations of BBQ and RBN2397.

(C) Data from NCI-H660 cells treated with the combinations of BBQ, FICZ and RBN2397.

Note: the treatment durations were 48 hrs for slower growing VCaP, CWR22Rv1 and NCI-H660, and 24 hrs for faster growing PC3-AR, PC3 and DU145.
