# Supplementary material for: Induction of PARP7 Creates a Vulnerability for Growth Inhibition by RBN2397 in Prostate Cancer Cells
Source: Cancer Res Commun. 2023 Apr 17;3(4):592–606. doi: 10.1158/2767-9764.CRC-23-0086 (PMC10108886; doi:10.1158/2767-9764.CRC-23-0086)
Supplement: Supplementary Figure 2 — BBQ and FICZ treatment activates AHR signaling in prostate cancer cells [file crc-23-0086-s03.pdf]

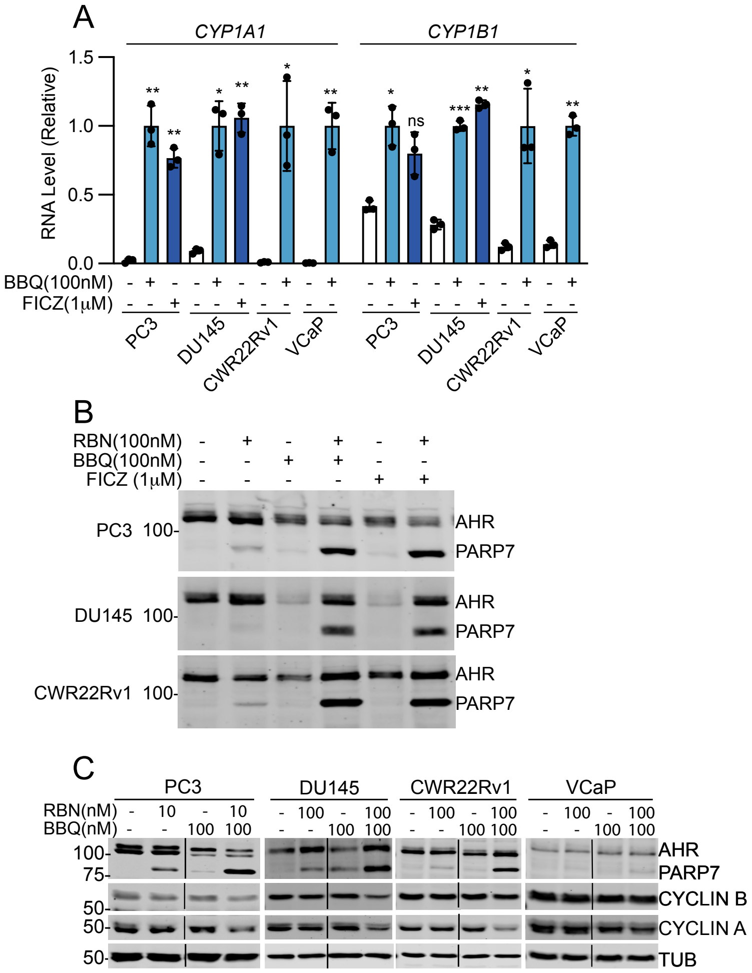

Supplementary Figure 2. BBQ and FICZ treatment activates AHR signaling in prostate cancer cells.
(A) *CYP1A1* and *CYP1B1* mRNA levels (normalized to*GUS*) are increased by BBQ and FICZ treatment in the prostate cancer cells. The experiments were done in triplicate. Error bars represent standard deviation. Statistical difference is between the vehicle control and AHR agonist-treated samples. ***, <0.001; **, <0.01; *, <0.05; ns, not significant.
(B) PARP7 protein levels are increased by the treatments with RBN2397 and AHR agonists.
(C) RBN2397 and BBQ combination treatments induce PARP7 protein levels but decrease CYCLIN A protein levels.
