# Supplementary material for: Induction of PARP7 Creates a Vulnerability for Growth Inhibition by RBN2397 in Prostate Cancer Cells
Source: Cancer Res Commun. 2023 Apr 17;3(4):592–606. doi: 10.1158/2767-9764.CRC-23-0086 (PMC10108886; doi:10.1158/2767-9764.CRC-23-0086)
Supplement: Supplementary Figure 3 — The growth inhibitory effect of RBN2397 in prostate cells is not dependent on TBK1 and JAK1/2 kinase activity [file crc-23-0086-s04.pdf]

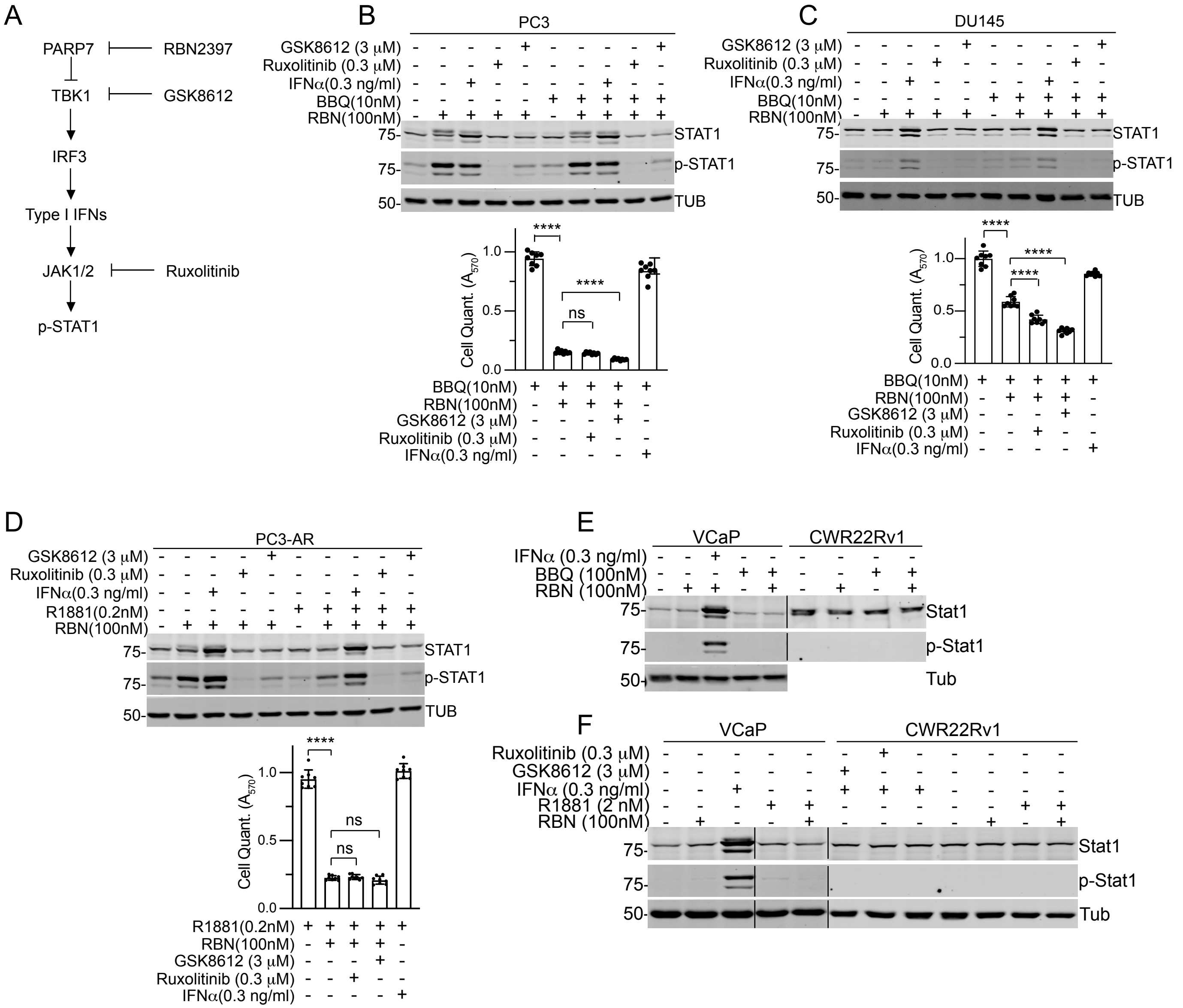

Supplementary Figure 3. The growth inhibitory effect of RBN2397 in prostate cells is not dependent on TBK1 and JAK1/2 kinase activity.

(A) The diagram shows a potential mechanism to link RBN2397 to JAK/STAT signaling in cell types including PC3 and PC3-AR cells. pathway of RBN2397 effects to STAT1 signaling in some cells, including PC3 and PC3-AR cells.

(B) Immunoblot detection of STAT1 and Phospho-STAT1 (left) and cell growth assays (right) in PC3 cells treated as indicated. Ruxolitinib (Ruxo, 0.3 μM) and GSK8612 (GSK, 3 μM) were used to inhibit TBK1 and JAK1/2, respectively, and IFNα (0.3 ng/ml) was used to activate JAK/STAT signaling. PARP7 induction and inhibition was mediated by addition of BBQ and RBN2397, respectively.

(C) Phospho-STAT1 levels are not altered detectably by RBN2397 treatment of DU145 cells, and the growth inhibitory effects of RBN2397 are not rescued by blocking TBK1 (GSK) and JAK1/2 (Ruxo) activity. The treatments in DU145 and the analysis were the same as described for panel A.

(D) The same treatments and analysis were as described for panel A, except R1881 was used to induce PARP7 in PC3-AR cells.

(E) Immunoblot analysis showing that RBN2397 does not promote STAT1 phosphorylation in VCaP and CWR22Rv1 cells in the presence or absence of BBQ.

(F) Immunoblot analysis showing that RBN2397 does not promote STAT1 phosphorylation in VCaP and CWR22Rv1 cells in the presence or absence of R1881.
