# Supplementary material for: Induction of PARP7 Creates a Vulnerability for Growth Inhibition by RBN2397 in Prostate Cancer Cells
Source: Cancer Res Commun. 2023 Apr 17;3(4):592–606. doi: 10.1158/2767-9764.CRC-23-0086 (PMC10108886; doi:10.1158/2767-9764.CRC-23-0086)
Supplement: Supplementary Figure 4 — RBN2397 inhibition of prostate cancer cell growth requires PARP7 over-expression [file crc-23-0086-s05.pdf]

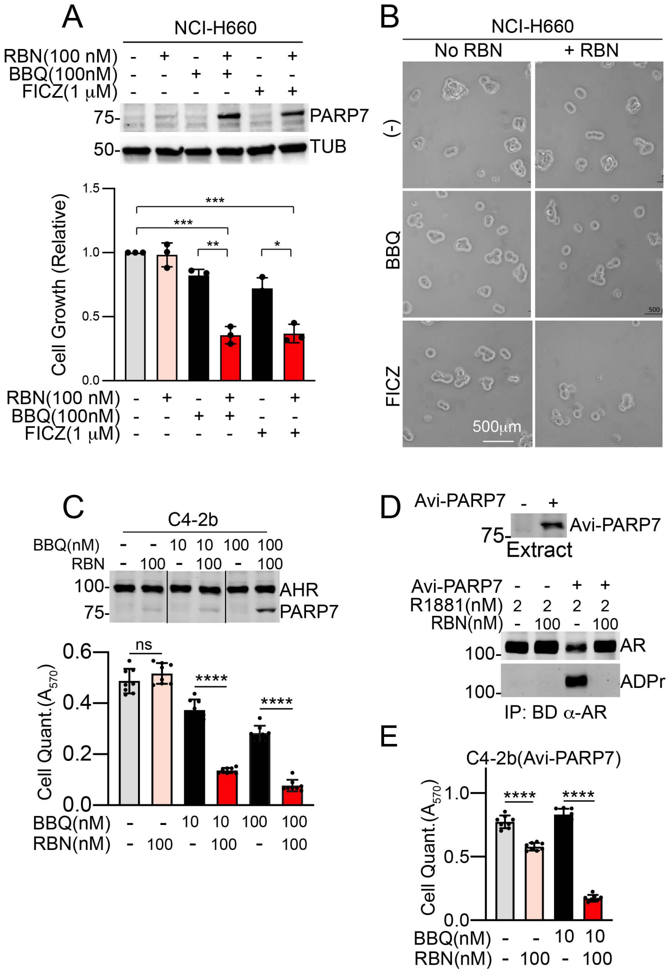

Supplementary Figure 4. RBN2397 inhibition of prostate cancer cell growth requires PARP7 over-expression.

(A) Effect of RBN2397 on NCI-H660 cells treated with AHR agonists BBQ or FICZ to induce PARP7. PARP7 levels are shown (upper panel). Each condition reflects five biological replicates. In A, C and E, error bars show the standard deviation, and ****, p<0.0001; ***, p<0.001; **, <0.01, *, p<0.05; ns, not significant.

(B) Phase contrast microscopy showing the morphology of cells using the treatments shown in (A).

(C) Effect of RBN2397 on C4-2b cell lines treated with AHR agonists BBQ. In C and E, each condition reflects eight biological replicates.

(D) Avi-tagged PARP7 (Avi-PARP7) over-expression in C4-2b cells (upper panel) characterized by immunoprecipitation, immunoblotting, and detection of AR ADP-ribosylation with Fl-Af1521.

(E) RBN2397 inhibits the growth of C4-2b(Avi-PARP7) cells in the absence of AHR agonist BBQ.
