# Supplementary material for: Induction of PARP7 Creates a Vulnerability for Growth Inhibition by RBN2397 in Prostate Cancer Cells
Source: Cancer Res Commun. 2023 Apr 17;3(4):592–606. doi: 10.1158/2767-9764.CRC-23-0086 (PMC10108886; doi:10.1158/2767-9764.CRC-23-0086)
Supplement: Supplementary Figure 5 — FACS analysis of NCI-H660 cells for detection of necrosis and apoptosis [file crc-23-0086-s06.pdf]

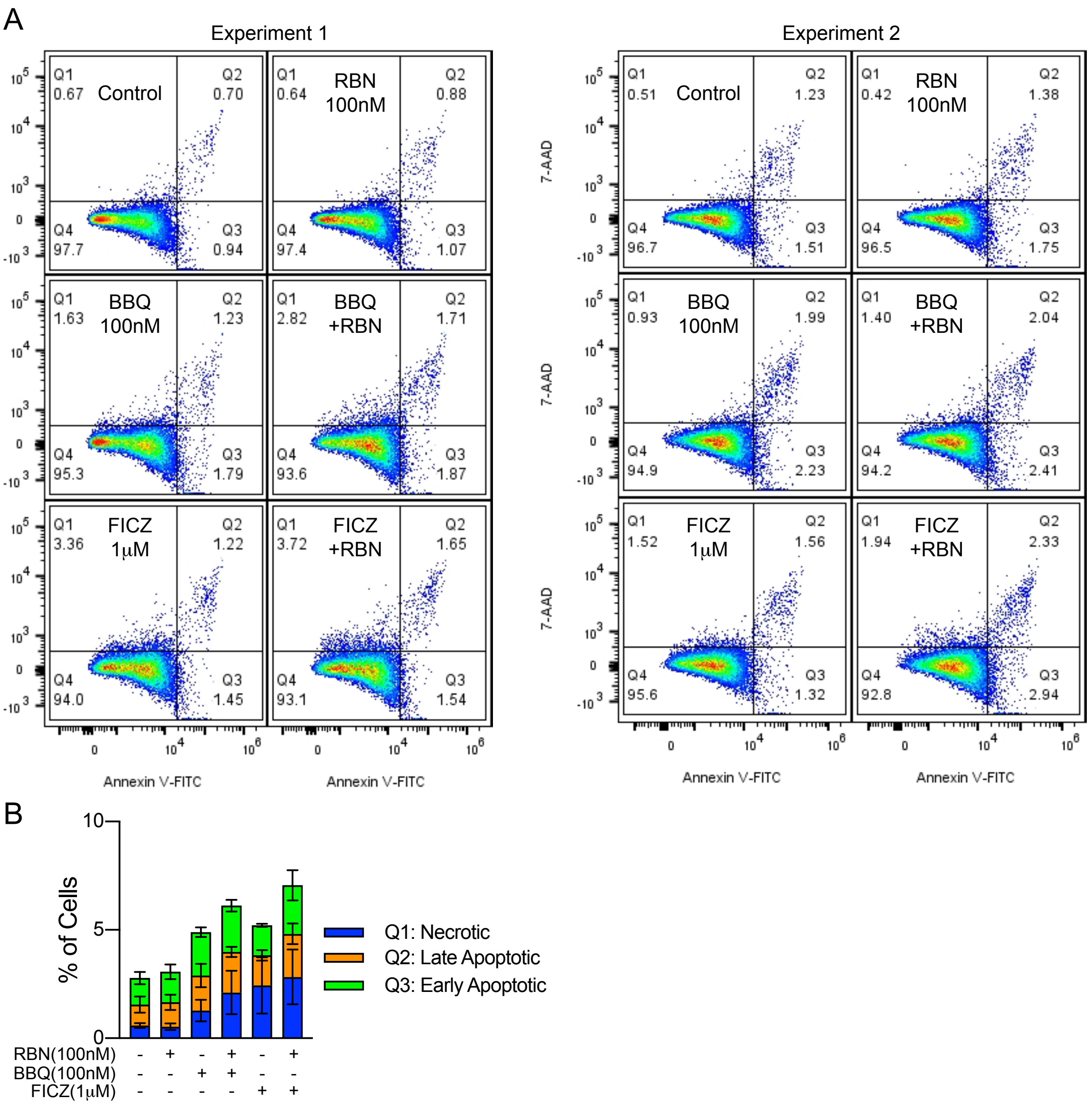

Supplementary Figure 5. FACS analysis of NCI-H660 cells for detection of necrosis and apoptosis.

(A) Cells were treated with BBQ, FICZ and RBN for 5 days, stained with Annexin V-FITC and 7-AAD, and examined by flow cytometry.

(B) Percentage of NCI-H660 cells in necrosis, late apoptosis and early apoptosis. Data from two experiments were combined and plotted as the mean and range.
