# Supplementary material for: Induction of PARP7 Creates a Vulnerability for Growth Inhibition by RBN2397 in Prostate Cancer Cells
Source: Cancer Res Commun. 2023 Apr 17;3(4):592–606. doi: 10.1158/2767-9764.CRC-23-0086 (PMC10108886; doi:10.1158/2767-9764.CRC-23-0086)
Supplement: Supplementary Figure 6 — Extendend analysis of PARP7 expression in prostate cancer [file crc-23-0086-s07.pdf]

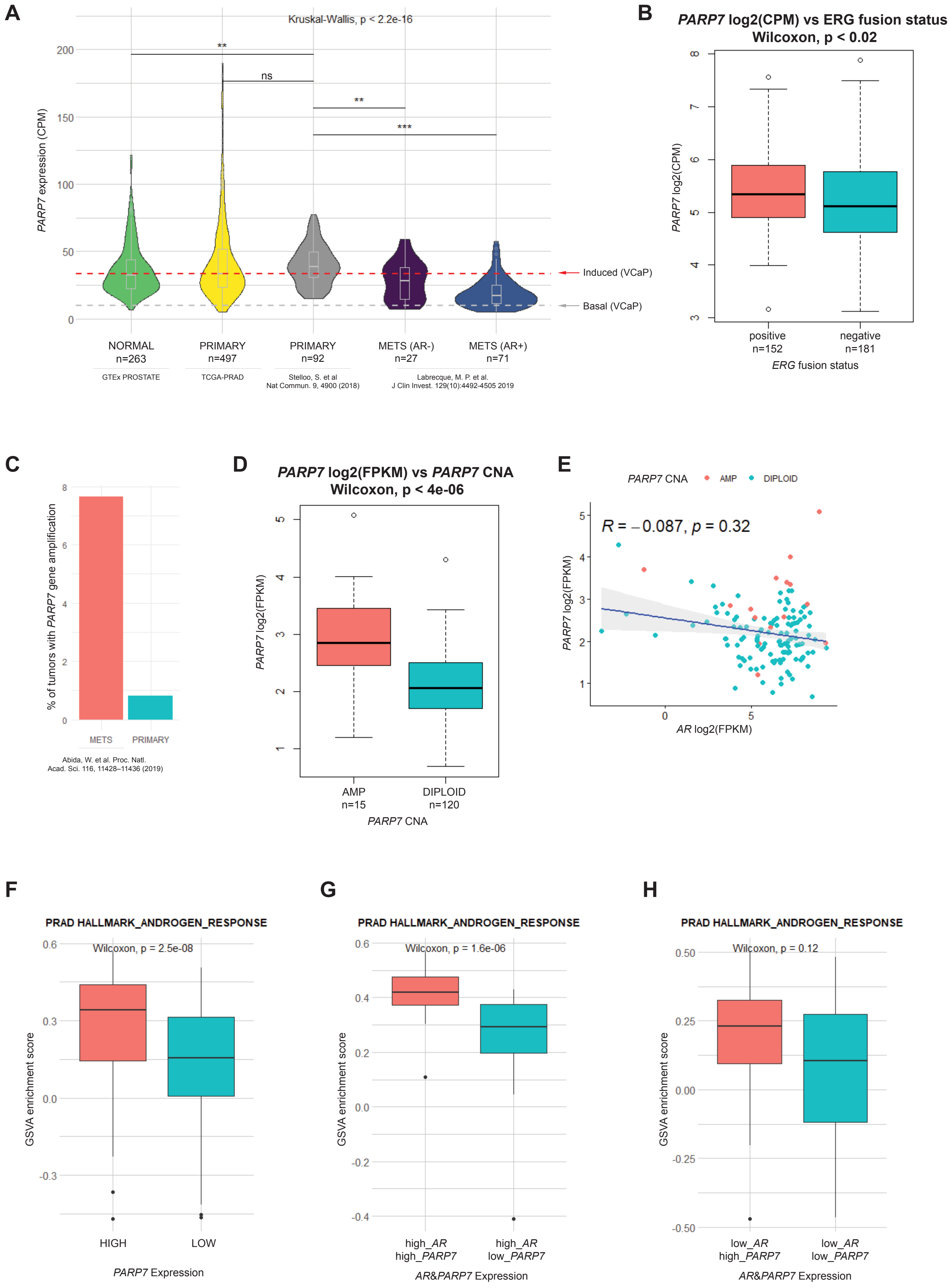
A
Kruskal-Wallis, p < 2.2e-16
PARP7 expression (CPM)
200
150
100
50
0
**
ns
**
***
Induced (VCaP)
Basal (VCaP)
NORMAL
n=263
PRIMARY
n=497
PRIMARY
n=92
METS (AR-)
n=27
METS (AR+)
n=71
GTEx PROSTATE
TCGA-PRAD
Stelloo, S. et al
Nat Commun. 9, 4900 (2018)
Labrecque, M. P. et al.
J Clin Invest. 129(10):4492-4505 2019
B
PARP7 log2(CPM) vs ERG fusion status
Wilcoxon, p < 0.02
PARP7 log2(CPM)
positive
n=152
negative
n=181
ERG fusion status
C
% of tumors with PARP7 gene amplification
METS
PRIMARY
Abida, W. et al. Proc. Natl.
Acad. Sci. 116, 11428–11436 (2019)
D
PARP7 log2(FPKM) vs PARP7 CNA
Wilcoxon, p < 4e-06
PARP7 log2(FPKM)
AMP
n=15
DIPLOID
n=120
PARP7 CNA
E
PARP7 CNA
AMP
DIPLOID
R = –0.087, p = 0.32
PARP7 log2(FPKM)
AR log2(FPKM)
F
PRAD HALLMARK_ANDROGEN_RESPONSE
Wilcoxon, p = 2.5e-08
GSVA enrichment score
HIGH
LOW
PARP7 Expression
G
PRAD HALLMARK_ANDROGEN_RESPONSE
Wilcoxon, p = 1.6e-06
GSVA enrichment score
high_AR
high_PARP7
high_AR
low_PARP7
AR&PARP7 Expression
H
PRAD HALLMARK_ANDROGEN_RESPONSE
Wilcoxon, p = 0.12
GSVA enrichment score
low_AR
high_PARP7
low_AR
low_PARP7
AR&PARP7 Expression

Supplementary Figure 6. Extended analysis of *PARP7* expression in prostate cancer

(A) Violin plots (boxplots inserted) with the *PARP7* expression data in Counts Per Million (CPM) from Figure 8A, with the additional *PARP7* expression data (grey shading), from Stelloo, S. et al. Nat Commun. 2018. *PARP7* expression is similar in the two primary prostate cancer tumor data sets. The red line indicates the *PARP7* level in VCaP cells induced by R1881 and found to sensitize cells to RBN2397-mediated growth inhibition, and the gray line shows the basal level of PARP7 in VCaP cells. P-values calculated for pairwise comparisons using the Wilcoxon Rank Sum test are indicated (***, <0.001; **, <0.01; *, < 0.05; ns, not significant). These comparisons were done only for additional dataset, since the rest is shown on the fig. 8A. All compared values come from the recount3 project.
(B) Boxplots comparing *PARP7* expression levels (CPM, log2 transformed) in *ERG* fusion positive and negative primary prostate cancers (TCGA-PRAD). P-value calculated using Wilcoxon Rank Sum test is indicated on the figure.
(C) Bar plot showing the percentage of tumors with *PARP7* gene amplification in primary prostate cancer tumors (TCGA-PRAD) and metastatic prostate cancer tumors (SU2C/PCF Dream Team, PNAS 2019). Data was acquired from cBioPortal (https://www.cbioportal.org/).
(D) Boxplots comparing *PARP7* expression level Fragments Per Kilobase Million, FPKM (log2 transformed) in metastatic prostate cancer tumors (SU2C/PCF Dream Team, PNAS 2019) with (AMP) and without (DIPLOID) *PARP7* gene amplification. Data was acquired from cBioPortal (https://www.cbioportal.org/). The data shown reflects the subset of tumors for which RNA-seq data is available. P-value calculated using Wilcoxon Rank Sum test is indicated on the figure.
(E) Scatter plot comparing expression of *PARP7* with AR (FPKM, log2 transformed) in metastatic prostate cancer tumors (SU2C/PCF Dream Team, PNAS 2019). Spearman correlation coefficient and the p-value are shown on the plot. Tumors with (AMP) and without (DIPLOID) *PARP7* gene amplification are labeled as indicated in the legend. Data was acquired from cBioPortal (https://www.cbioportal.org/).
(F) Boxplots comparing enrichment scores for the HALLMARK_ANDROGEN_RESPONSE gene set, calculated using the GSVA method between high *PARP7* expression (top quartile, n=124) and low *PARP7* expression (bottom quartile, n=124) in primary prostate cancer tumors (TCGA-PRAD). P-value calculated using Wilcoxon Rank Sum test is indicated on the figure.
(G) Boxplots comparing enrichment scores for the HALLMARK_ANDROGEN_RESPONSE gene set, calculated using the GSVA method between high *AR* expression (top quartile), high *PARP7* expression (top quartile) (n=32) and with high *AR* expression (top quartile), low *PARP7* expression (bottom quartile) (n=26) in primary prostate cancer tumors (TCGA-PRAD). P-value calculated using Wilcoxon Rank Sum test is indicated on the figure.
(H) Boxplots comparing enrichment scores for the HALLMARK_ANDROGEN_RESPONSE gene set, calculated using the GSVA method between low *AR* expression (bottom quartile), high *PARP7* expression (top quartile) (n=26) and with low *AR* expression (bottom quartile), low *PARP7* expression (bottom quartile) (n=46) in primary prostate cancer tumors (TCGA-PRAD). P-value calculated using Wilcoxon Rank Sum test is indicated on the figure.
